# INTERNATIONAL ENVIRONMENTAL HEALTH: Invasion of the Bedbugs

**DOI:** 10.1289/ehp.118-a429

**Published:** 2010-10

**Authors:** John Manuel

**Affiliations:** **John Manuel** of Durham, NC, is a regular contributor to *EHP* and the author of *The Natural Traveler Along North Carolina’s Coast* and *The Canoeist*.

The invaders have arrived, and our chemical weapons are practically useless to stop them. They have reached every major city, threatening students in college dorms and taking over stores, apartments, and hotels across the country. People are begging authorities for help and, increasingly, taking matters into their own hands. What sounds like a script for a science fiction movie is, in fact, the reality of the fight against bedbugs.

Bedbugs have been annoying humans since ancient times. They are mentioned in medieval European texts and in classical Greek writings back to the time of Aristotle.^1^ Following the introduction of the pesticide DDT in the 1950s, bedbugs virtually disappeared in the United States. But now they are making a comeback. Anecdotes about infestations can be found in blogs and newspapers in virtually every major city.

The extent of the problem is suggested by a survey conducted by the National Pest Management Association, Inc. (NPMA) and the University of Kentucky, which had respondents from 43 countries.^2^ Of 521 responding U.S. pest management companies, 95% reported encountering a bedbug infestation in the past year. Similar numbers were reported for Canada (98%), Europe (92%), and Africa/Middle East (90%). By comparison, before 2000 only 25% of U.S. survey respondents reported bedbug infestations, according to a 26 July 2010 press release from the NPMA.^3^

The common bedbug (*Cimex lectularius*) is about a quarter-inch long with a reddish-brown, oval, flattened body. It feeds solely on the blood of warm-blooded animals, preferably humans. Bedbugs usually bite people at night while they are sleeping. Some people show little or no reaction to the bites. Others develop welts that cause severe itching and, in rare cases, anaphylaxis. Anecdotal evidence indicates anxiety about bedbugs can be as bad as an actual infestation, even causing “delusional parasitosis,” a condition in which people mistakenly believe they are infected with parasites.

Bedbugs are not known to transmit disease except for possible associations with hepatitis B^4^ and Chagas disease.^5^ They are classified by the U.S. Environmental Protection Agency (EPA) as “a pest of significant public health importance” under the Federal Insecticide, Fungicide, and Rodenticide Act.^6^

Experts are not certain of the cause for the bedbug resurgence. The increased movement of people domestically and internationally is thought to be one factor. Another is the resistance bedbugs have developed to pesticides. “Bedbugs have been treated so many times, they have developed a resistance to commercially available products allowed for use by the EPA,” says Dini Miller, an associate professor of entomology at Virginia Polytechnic Institute and State University.

Propoxur (sold as Baygon®) is one commercially available chemical that is still effective in killing bedbugs. This carbamate pesticide is widely used in commercial settings in the United States and was once approved for use in residential environments. However, propoxur is toxic to humans if ingested. Pesticide manufacturers, recognizing that indoor use of certain pesticides would not pass the more stringent testing requirements under the Food Quality Protection Act (FQPA) of 1996, agreed to drop their registration of propoxur for residential use.

The removal of propoxur and similar pesticides has left pest management companies with a limited array of tools to combat infestations. Steamers and rapid freezing equipment will kill bedbugs on contact. But the insects are experts at hiding, and repeated treatments are required to be effective. At an NPMA-estimated cost of $500–1,200 per session, such treatments are unaffordable for many people.

State and local government officials say they are being overwhelmed with complaints of bedbug infestations. They also are hearing of widespread misuse of pesticides. Miller visited one home in which the resident had set off 30 “bug bombs” at once.^7^ Another blew the walls out of his apartment after setting off a bug bomb and failing to turn off the pilot light (aerosol propellants can ignite in an enclosed room). Officials in the state of Ohio have become so concerned that they have asked the EPA to grant them an exemption under Section 18 of the FPQA, allowing them to use propoxur in residential environments.

That exemption request is still pending, according to spokeswoman Kaleigh Frazier of the Ohio Department of Agriculture. “The EPA has not issued a formal denial on this request,” she says. “We continue to work with the EPA to get this matter resolved.” However, EPA scientists believe the use could present an unacceptable risk to children who might be exposed to propoxur in and around rooms being treated for bedbugs, according to Dale Kemery, a press officer with the EPA.

In August 2010, the EPA and the Centers for Disease Control and Prevention issued a joint statement promoting an integrated pest management (IPM) approach to bedbug control.^8^ IPM includes heat treatment, vacuuming, nonchemical pesticides (such as diatomaceous earth), and “judicious use” of chemical pesticides.^9^ The EPA says it is actively working with industry and researchers to develop new compounds (or new uses of existing compounds) to control bedbugs.

That can’t come soon enough for Miller, who has requested government funding to study the bedbug’s genetic and mechanical resistance mechanisms. “Our hope is to eventually manipulate those mechanisms,” Miller says. “In the meantime, we are essentially defenseless.”

## Figures and Tables

**Figure:**
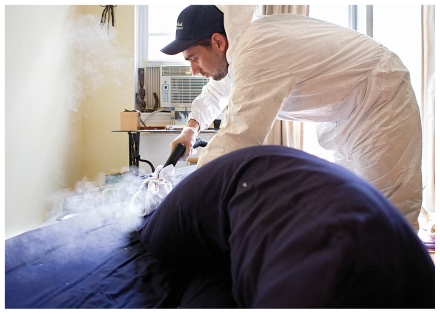
An exterminator fumigates a Queens, New York apartment for bedbugs, 28 July 2010.
